# Assessing safety and efficacy of TNFi treatment in late onset ankylosing spondylitis: a TURKBIO registry study

**DOI:** 10.1038/s41598-024-65180-4

**Published:** 2024-06-20

**Authors:** Sadettin Uslu, Semih Gulle, Gercek Sen, Ayse Cefle, Sema Yilmaz, Sinem Burcu Kocaer, Tuba Yuce Inel, Suleyman Serdar Koca, Servet Yolbas, Mehmet Akif Ozturk, Soner Senel, Nevsun Inanc, Huseyin Ediz Dalkilic, Ozgul Soysal Gunduz, Abdurrahman Tufan, Servet Akar, Ahmet Merih Birlik, Ismail Sari, Nurullah Akkoc, Fatos Onen

**Affiliations:** 1 Division of Rheumatology, Celal Bayar University School of Medicine, Manisa, Turkey; 2 Division of Rheumatology, Batman Training and Research Hospital, Batman, Turkey; 3 Division of Rheumatology, Dokuz Eylül University School of Medicine, İzmir, Turkey; 4 Division of Rheumatology, Kocaeli University School of Medicine, Kocaeli, Turkey; 5 Division of Rheumatology, Selcuk University School of Medicine, Konya, Turkey; 6 Division of Rheumatology, Faculty of Medicine, Izmir University of Economics, Izmir, Turkey; 7 Division of Rheumatology, Bozyaka Training and Research Hospital, Izmir, Turkey; 8 Division of Rheumatology, Fırat University School of Medicine, Elazığ, Turkey; 9 Division of Rheumatology, Inonu University School of Medicine, Malatya, Turkey; 10 Division of Rheumatology, Gazi University School of Medicine, Ankara, Turkey; 11 Division of Rheumatology, Erciyes University School of Medicine, Kayseri, Turkey; 12 Division of Rheumatology, Marmara University School of Medicine, Istanbul, Turkey; 13 Division of Rheumatology, Uludağ University School of Medicine, Bursa, Turkey; 14 Division of Rheumatology, Izmir Kâtip Celebi University School of Medicine, İzmir, Turkey

**Keywords:** Ankylosing spondylitis, Late-onset ankylosing spondylitis, TNFi, 45 years, TURKBIO, Medical research, Rheumatology, Rheumatic diseases

## Abstract

Clinical data on the use of tumour necrosis factor inhibitors (TNFi) in late-onset ankylosing spondylitis (LoAS) are limited. The present study aimed to evaluate efficacy, safety, and treatment adherence associated with the initial use of TNFi therapy in biologic naive patients diagnosed with LoAS. Patients whose age of onset was ≥ 45 years and < 45 years were classified as having LoAS and YoAS, respectively, based on the age of symptom onset. There were 2573 patients with YoAS and 281 LoAS. Baseline disease activity measures were similar between the groups. No significant differences were seen between the two groups in response to treatment and in remaining on the first TNFi at 6, 12 and 24 months. In the LoAS group, the analysis showed that TNFi discontinuation was linked to VAS pain score (HR 1.04; 95% CI 1.01–1.06). Patient groups had similar rates of adverse events (YoAS: 8.7% vs. LoAS: 11.7%). In both biologic naive LoAS and YoAS patients, the study showed that the initial TNFi therapy was equally effective and safe.

## Introduction

The most common and prototypical form of spondyloarthritis (SpA), ankylosing spondylitis (AS), is characterized by axial involvement and radiographic evidence of sacroiliitis according to the modified New York criteria (mNY)^1^. AS is recognized for its early onset, with symptoms typically manifesting in the second or third decade of life. It is rare for symptoms to appear after the age of 45^2^. Late onset AS (LoAS) is estimated to occur in approximately 3.5–13.8% of all cases of AS^3,4^.

Defining LoAS still lacks consensus. Studies have used different criteria, with some defining LoAS as an onset age of ≥ 50 years, while others consider ≥ 40 and ≥ 45 years as indicators of late onset. Moreover, the late onset spectrum of spondyloarthritis (SpA) extends to include late onset peripheral SpA and late onset psoriatic arthritis (PsA)^3,5,6^. A recent study also explored late onset non-radiographic axial SpA (nr-AxSpA) patients^7^. There are different grouping recommendations for patients diagnosed with AS according to the age of onset of symptoms. In studies, the cut-off age according to the onset of symptoms has generally been chosen as 50 years of age. However, in ASAS consensus reports, the LoAS definition has come to the fore in patients who describe symptoms after the age of 45, since the onset of symptoms before the age of 45 is included in the criterion sets^8^.

While there are guidelines on the use of sulfasalazine and non-steroidal anti-inflammatory drugs (NSAIDs) in elderly or LoAS patients^9^. There are not many studies in the literature on the usage of TNF inhibitors (TNFi)^9,10^.

The primary objective of this study was to conduct a comprehensive evaluation of the efficacy, safety, and treatment adherence associated with the initial use of TNFi therapy in biologic naive patients diagnosed with LoAS, and to compare these outcomes with patients with earlier-onset symptoms, known as young onset AS (YoAS). Additionally, an in-depth analysis encompassed a comparative exploration of both demographic and clinical characteristics among these distinct patient groups.

## Methods

### Patient population and data collection

The Turkish Biological (TURKBIO) Database provided the data for this study, established in 2011 as the Turkish counterpart of the Danish DANBIO Rheumatological Database^11^. This extensive nationwide registry is primarily dedicated to collecting and analysing data from patients with rheumatic diseases being treated with biologic and targeted synthetic disease-modifying antirheumatic drugs (bDMARDs and tsDMARDs). The subjects of the current research were biologic naïve individuals who started their first TNFi medication and satisfied the mNY criteria for AS. Patients who met the study eligibility criteria and were enrolled in our study from 2011 to 2021 were included. In many studies, the cut-off age for symptom onset has generally been chosen as 50 years of age^10^. In this study, we planned to take the cut-off value as 45 years of age because the ASAS classification criteria include symptom onset before the age of 45^12^. Based on the age at which the patients' symptoms first appeared, they were further divided into two groups: YoAS (symptom onset at < 45 years) and LoAS (symptom onset at ≥ 45 years).

### Demographic and clinical features

Several demographic, clinical, and laboratory features, including age, sex, time since diagnosis, smoking status, body mass index, human leukocyte antigen B27 (HLA-B27) status, and treatment strategies (TNFi and co-medications with csDMARDs, NSAIDs, and glucocorticoids) were assessed in LoAS patients and compared with those in the YoAS group. The web-based registry of patients with rheumatic diseases receiving biological DMARDs was used as the data source in the study. Exclusion criteria were as follows: (1) patients without follow-up data and (2) patients who withdrew informed consent.

### Disease follow-up assessments

Patients with AS had their disease activity evaluated using a number of standardized metrics, including the Bath Ankylosing Spondylitis Disease Activity Index (BASDAI)^13^ and the Ankylosing Spondylitis Disease Activity Score (ASDAS)^14^. Additionally, the Bath Ankylosing Spondylitis Functional Index (BASFI)^15^ was employed to evaluate functional status, and the Health Assessment Questionnaire-Spondylitis (HAQ-S) was utilized to gauge health-related quality of life^16^. These assessments were carried out at baseline and at subsequent intervals of 6-, 12-, and 24-months following treatment initiation. The 6-month assessment was scheduled between 90 and 270 days after baseline, the 12-month assessment between 271 and 545 days, and the 24-month assessment between 546 and 910 days.

### Treatment response

Various disease activity measures, including ASDAS-CRP, ASDAS-inactive disease (ASDAS-ID), ASDAS-clinically important improvement (ASDAS-CII), ASDAS-Major improvement (ASDAS-MI), The Assessment of Spondyloarthritis International Society responses (ASAS 20/40)^17^, and BASDAI50^18^, were assessed at 6, 12, and 24 months. ASDAS-ID is the presence of ASDAS < 1.3. ASDAS criteria for improvement were ≥ 1.1 units for ASDAS-CII and ≥ 2.0 units for ASDAS-MI.

### Retention rates

The number of days between each patient's documented date of treatment beginning and date of treatment cessation was used to establish the treatment duration. When a new bDMARD was entered into the registry, treatment without a recorded stop date was considered to have been stopped, with the stop date being the date of the subsequent start of the bDMARD. The treatment periods were regarded as a single period if the same medication was resumed within 3 months of the documented treatment stop date and no other bDMARD was reported in between. Retention rates were calculated by looking at the percentage of patients who remained on TNFi at 6, 12 and 24 months after starting treatment. The dates of (1) data extraction; (2) death; or (3) registry follow-up termination, whichever occurred first, were used to censor observations; and (4) treatment withdrawal for reasons other than adverse events (AE) and lack of efficacy (LOE), such as remission or other plans to become pregnant, were used to exclude observations. Reasons for discontinuation, including LOE and AE, were evaluated and compared.

### Compliance with ethical standards

The TURKBIO database project was approved as a phase IV observational study by the Turkish Ministry of Health Drug Regulatory Agency and the Local Ethics Committee (Decision No: 20.06.2013/253). Participants gave written informed consent for TURKBIO registry, and an additional consent was exempted due to the retrospective nature of this study. The Declaration of Helsinki's guiding principles were followed in the conduct of the study.

### Statistical analysis

SPSS 25.0 (IBM Corporation, Armonk, New York, USA) was used for data analysis and Shapiro–Wilk, Kolmogrov Smirnoff tests were used for normal distribution analysis. According to the normal distribution results, parametric tests were used to analyse the results showing normal distribution and non-parametric tests were used to analyse the variables not showing normal distribution. Quantitative variables were expressed as mean ± SD (Standard Deviation) and median (Minimum/Maximum) and categorical variables were expressed as n (%). Drug retention rate analyzes were calculated using the Kaplan–Meier method. The log-rank test was used to compare survival rates among the matched study groups created. The association of various factors with the risk of TNFi discontinuation in AS patients was assessed by Cox regression analysis. Variables were analysed at 95% confidence level and a *p* value less than 0.05 was considered significant.

### Ethics

The TÜRKBIO database project has been approved as a phase IV observational study by the Turkish Ministry of Health Drug Regulatory Agency and also by the Gaziantep University Ethics Committee (Decision No: 20.06.2013/253 Date: 20.06.2013). The coordination center of Dokuz Eylül University TURKBIO Rheumatology Department is also located in this city. The Scientific Committee consists of a physician responsible for each participating center. Participants gave written informed consent for TURKBIO registry, and an additional consent was exempted due to the retrospective nature of this study. The study was conducted in accordance with the principles of the Declaration of Helsinki.

## Results

### Patient details

A total of 2854 patients with follow-up data spanning at least a year were included in the research. Of these, 2573 (90.1%) fell into the YoAS group, and 281 (9.9%) were in the LoAS group. The mean age for YoAS patients was 42 ± 1 years, while LoAS patients had a mean age of 61 ± 7 years (*p* < 0.001). In the LoAS group, there were more female patients (59.1%) compared to the YoAS group (36.9%) (*p* < 0.001). LoAS patients had a significantly shorter time since diagnosis and disease duration compared to YoAS patients (*p* < 0.001). A higher proportion of patients in the LoAS group were never smokers (55.2% vs. 38.8%, *p* < 0.001), and HLA-B27 positivity was lower in the LoAS group (50.8% vs. 66.8%, *p* < 0.001). The distribution of peripheral arthritis, enthesitis, dactylitis, and extra-musculoskeletal findings (uveitis, psoriasis, and inflammatory bowel disease) was similar in both groups. There were no differences in family history of SpA and related disorders between the groups. Baseline CRP (14.57 ± 22.28 vs. 15.94 ± 24.7), BASDAI (34.6 ± 23.21 vs. 36.05 ± 22.67) and ASDAS-CRP mean values (2.73 ± 1.21 vs. 2.81 ± 1.14) were similar in both groups (*p* > 0.05). However, baseline ESR (23.28 ± 20.59 vs. 30.05 ± 21.67), BASMI (29.76 ± 23.13 vs. 37.72 ± 23.28) and BASFI (25.61 ± 23.02 vs. 30.07 ± 23.13) were found to be higher in the LoAS group (*p* < 0.001, *p* = 0.002 and *p* = 0.001, respectively). Table 1 summarized the clinical and laboratory characteristics of the patients.

**Table 1:** Demographic features of YoAS and LoAS patients.

Baseline characteristics	YoAS		LoAS		*p* value
	n*	Mean ± SD	n*	Mean ± SD	
Age, years	2573	42 ± 10	281	61 ± 7	**< 0.001**
Time since diagnosis (year)	2538	4 ± 6	277	2 ± 3	**< 0.001**
Disease duration (year)	2570	11 ± 8	281	7 ± 5	**< 0.001**
Diagnosis delay (year)	2538	7 ± 4	277	5 ± 3	0.443
Education	1755	10 ± 4	202	7 ± 4	**< 0.001**
C-reactive protein (mg/dL)	2473	14.57 ± 22.28	277	15.94 ± 24.7	0.132
Erythrocyte sedimentation rate (mm/hr)	2447	23.28 ± 20.59	269	30.05 ± 21.67	**< 0.001**
BASDAI (baseline)	2480	34.6 ± 23.21	268	36.05 ± 22.67	0.268
BASMI (baseline)	737	29.76 ± 23.13	81	37.72 ± 23.28	**0.002**
BASFI (baseline)	2455	25.61 ± 23.02	267	30.07 ± 23.13	**0.001**
ASDAS-CRP (unit)	2144	2.73 ± 1.21	250	2.81 ± 1.14	0.282
VAS global	2460	45.11 ± 29.37	279	48.94 ± 27.6	0.064
VAS pain	2470	47.84 ± 29.65	279	51.76 ± 26.76	0.073

	n*	n (%)	n*	n (%)	*p* value
Gender, male	2573	1623 (63.1)	281	115 (40.9)	**< 0.001**
HLA B27 (+)	1690	1129 (66.8)	187	95 (50.8)	**< 0.001**
Smoking status	2325		252		**< 0.001**
Never		884 (38)		139 (55.2)	
Current/occasionally		1061 (45.6)		50 (19.8)	
Ex-smoker		380 (16.4)		63 (25)	
Extraarticular manifestations					
Uveitis	2509	226 (9)	276	16 (5.8)	0.072
Peripheral arthritis	660	227 (34.4)	83	32 (38.6)	0.453
Enthesitis	470	179 (38.1)	47	16 (34)	0.586
Inflammatory bowel disease	2509	82 (3.3)	276	10 (3.6)	0.754
Psoriasis	2509	50 (2)	276	5 (1.8)	0.837
Dactylitis	471	49 (10.4)	47	6 (12.8)	0.625
Family history	595	188 (31.6)	69	22 (31.9)	0.961
Concomitant csDMARD	2573	–	281	–	–
Methotrexate		135 (5.2)		23 (8.2)	**0.041**
Sulphasalasine		279 (10.8)		49 (17.4)	**0.001**
TNFi treatment	2573	–	281	–	–
Adalimumab		770 (30.0)		87 (30.1)	
Certolizumab		320 (12.4)		36 (12.8)	
Etanercept		690 (26.8)		75 (26.7)	0.942
Golimumab		425 (16.5)		41 (14.5)	
Infliximab		368 (14.3)		42 (14.9)	

Significant values are in [bold].

n* number of patients with available data from registry, *CRP* C-reactive protein, *YoAS* young onset ankylosing spondylitis (symptom onset < 45 years), *LoAS* late onset ankylosing spondylitis (symptom onset ≥ 45 years), *BASDAI* bath ankylosing spondylitis disease activity index, *BASMI* bath ankylosing spondylitis metrology index, *BASFI* bath ankylosing spondylitis functional index, *ASDAS* ankylosing spondylitis disease activity score, *csDMARD* conventional synthetic disease modifying anti rheumatic drug, *VAS* visual analog scale, *n* number, *%* percent, *SD* standard deviation.

### Treatment preference and response

In both groups, the distribution of TNFi usage was comparable (*p* = 0.846). Patients with LoAS were more likely to concurrently utilize csDMARDs, such as methotrexate and sulfasalazine (*p* = 0.041 and *p* = 0.001, respectively). At 6, 12, and 24 months, several therapy response markers were compared between the groups. Only the response rate for ASDAS-ID (ASDAS-CRP < 1.3) at 6 months was lower in the LoAS group compared to YoAS (*p* = 0.005), but this difference disappeared at the 12- and 24-month follow-ups. Other treatment response indexes at these time points were similar. There was no discernible difference between the groups in terms of patients achieving ASDAS-MI, ASDAS-CII, BASDAI-40, BASDAI-50, ASAS20 and ASAS40 at 6, 12 and 24 months. Table 2 provides an overview of patient response rates following therapy.

**Table 2:** Disease activity score changes of patients.

	YoAS		LoAS		*p* value
	n*	n (%)	n*	n (%)	
ASDAS < 1.3					
6th months	1436	502 (35)	163	39 (23.9)	**0.005**
12th months	1205	444 (36.8)	123	46 (37.4)	0.904
24th months	925	356 (38.5)	89	31 (34.8)	0.498
ASDAS-MI					
6th months	1295	308 (23.8)	153	29 (19)	0.181
12th months	1017	257 (25.3)	110	27 (24.5)	0.868
24th months	728	206 (28.3)	77	24 (31.2)	0.596
ASDAS-CII					
6th months	1295	568 (43.9)	153	62 (40.5)	0.431
12th months	1017	473 (46.5)	110	54 (49.1)	0.606
24th months	728	354 (48.6)	77	40 (51.9)	0.579
BASDAI < 40					
6th months	1563	1386 (88.7)	175	149 (85.1)	0.168
12th months	1267	1144 (90.3)	131	122 (93.1)	0.290
24th months	986	906 (91.9)	97	94 (96.9)	0.076
BASDAI 50					
6th months	1528	820 (53.7)	170	78 (45.9)	0.054
12th months	1227	744 (60.6)	127	77 (60.6)	0.999
24th months	946	614 (64.9)	92	63 (68.5)	0.492
ASAS 20					
6th months	1504	811 (53.9)	168	82 (48.8)	0.208
12th months	1203	664 (55.2)	125	74 (59.2)	0.391
24th months	898	497 (55.3)	91	59 (64.8)	0.082
ASAS 40					
6th months	1506	785 (52.1)	168	76 (45.2)	0.090
12th months	1204	646 (53.7)	125	72 (57.6)	0.400
24th months	898	487 (54.2)	91	59 (64.8)	0.053

Significant values are in [bold].

n* Number of patients with available data from registry, *CRP* C-reactive protein, *YoAS* young onset ankylosing spondylitis (symptom onset < 45 years), *LoAS* late onset ankylosing spondylitis (symptom onset ≥ 45 years), *BASDAI* bath ankylosing spondylitis disease activity index, *ASAS* Assessment of Spondyloarthritis International Society, *ASDAS* ankylosing spondylitis disease activity score, *ASDAS-CII* ASDAS clinically important improvement, *ASDAS-MI* ASDAS major improvement, *n* number, *%* percent.

### Drug retention

**Figure 1:**
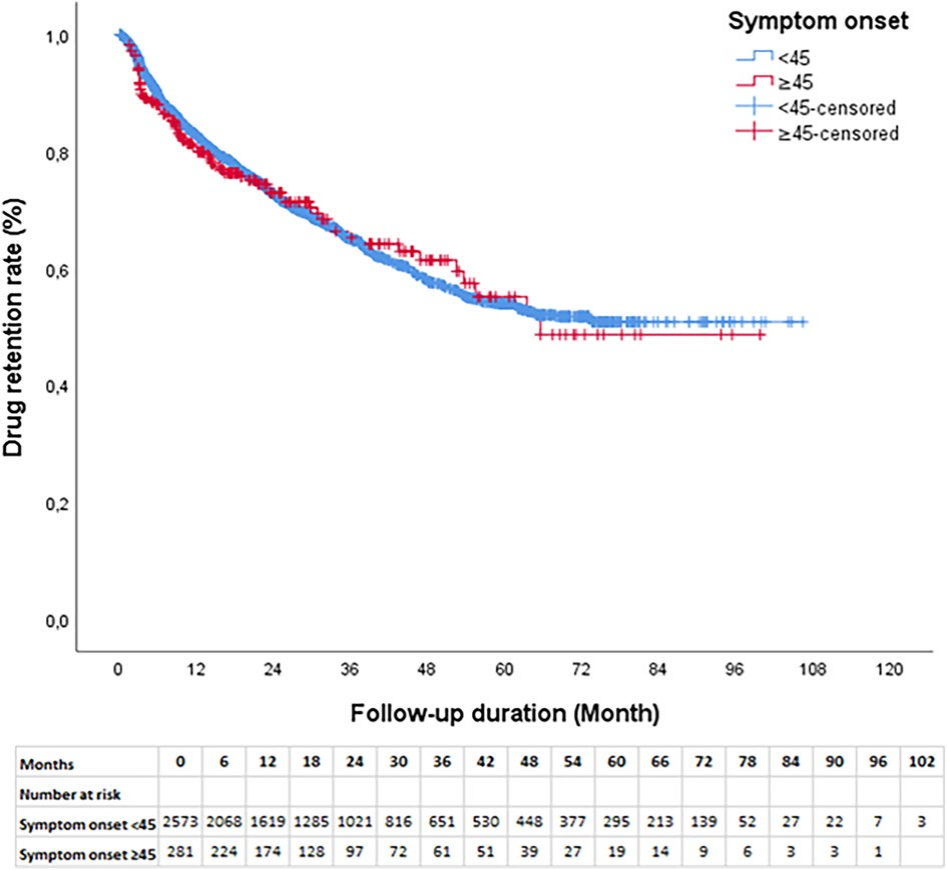
First TNFi drug survival graphic in YoAS and LoAS groups.

As shown in Fig. 1, the two groups' TNFi therapy retention rates did not differ significantly from one another (*p* = 0.789). Of the 2854 patients that were observed for 24 months, 822 (28.8%) of them stopped receiving therapy. In the YoAS group, the retention rates stood at 81.1% at 12 months and 72.9% at 24 months. In the LoAS group, the retention rates were 83.1% at 12 months and 72.9% at 24 months, and there were no substantial differences when compared to YoAS (*p* > 0.05). The most prevalent reason for discontinuation at the 24-month mark was inefficacy, accounting for 44.9% in the YoAS group and 50.7% in the LoAS group, followed by AEs (8.7% in YoAS and 11.7% in LoAS) and remission (1.6% in YoAS and 1.3% in LoAS) as detailed in Table 3. Twelve (1.6%) patients in the YoAS group and 1 (1.3%) patient in the LoAS group were considered in remission due to inactive disease and continued treatment without TNFi. These patients were excluded from the analysis of drug survival. Reasons for discontinuation due to drug ineffectiveness and AE were evaluated.

**Table 3:** Reasons for discontinuation of biologic therapies in patients using TNFi.

	YoAS (n = 2573)	LoAS (n = 281)
	n (%)	n (%)
Drug discontinuation rate	745 (28.9)	77 (27.4)
Reason for discontinuation		
Remission	12 (1.6)	1 (1.3)
Inefficacy	334 (44.9)	39 (50.7)
Adverse events	65 (8.7)	9 (11.7)
Severe infections	9 (1.2)	1 (1.3)
Allergic reactions	9 (1.2)	1 (1.3)
Tuberculosis	10 (1.3)	2 (2.6)
Malignancy	6 (0.8)*	3 (3.9)**
Others severe events***	31 (4.2)	2 (2.6)
Other reasons****	334 (44.8)	28 (36.3)

*Plasmacytosis (n = 1), lung cancer (n = 1), basal cell carcinoma of skin in situ (n = 1), acute leukaemia (n = 1), renal malign neoplasm (n = 1), bladder neoplasm (n = 1).

**Prostate cancer (n = 1), thyroid papillary carcinoma (n = 1), benign neoplasm of thymus (n = 1).

***Paradoxical psoriasis, sarcoidosis, iridocylitis, retinal vasculitis, peripheral neuropathy, pancytopenia, acute pancreatitis, autoimmune hepatitis.

****Follow-up loss, planned or confirmed pregnancy, costs or reimbursement issues.

### Safety

A total of 9 serious AEs were reported during follow-up in LoAS patients, constituting 11.7% of that group, while in the YoAS group, 65 serious AE were reported, accounting for 8.7%. Among these events, severe infections were the most frequently encountered, with rates of 1.3% in LoAS and 1.2% in YoAS. Allergic reactions were also reported at similar rates, 1.3% in LoAS and 1.2% in YoAS. Tuberculosis was observed in 2 patients (2.6%) in the LoAS group and 10 patients (1.3%) in the YoAS group. Malignancy was reported in 3 patients (3.9%) in the LoAS group and 6 patients (0.8%) in the YoAS group, as detailed in Table 3.

### Factors influencing TNFi retention in all AS patients and by age groups

An analysis using Cox regression revealed that several factors were associated with the risk of TNFi discontinuation in all AS patients. These factors included female sex (HR 0.71; 95% CI 0.60–0.84, *p* < 0.001), higher baseline BASFI (HR 1.01; 95% CI 1.00–1.01), HLA-B27 negativity (HR 0.72; 95% CI 0.61–0.86), and VAS pain score (HR 1.01; 95% CI 1.00–1.01). In the LoAS group, the analysis showed that TNFi discontinuation was linked to VAS pain score (HR 1.04; 95% CI 1.01–1.06). Tables 4 and 5 summarize factors affecting TNFi retention in all patients and by age groups.

**Table 4:** Factors associated with first TNFi discontinuation in all AS patients.

	AS patients (All) (n = 2854)			
	Univariate analysis		Multivariable analysis	
	Hazard ratio (95% CI)	*p* value	Hazard ratio (95% CI)	*p* value
Age, years (median)	1.00 (0.99–1.01)	0.923		
Male (male vs. female)	0.65 (0.57–0.74)	**< 0.001**	0.71 (0.60–0.84)	**< 0.001**
Symptom onset, years	1.00 (1.00–1.01)	0.883	1.00 (1.00–1.01)	0.883
Current smoker	0.90 (0.78–1.04)	**0.163**		
HLA B27 positivity	0.71 (0.60–0.83)	**< 0.001**	0.72 (0.61–0.86)	**< 0.001**
Elevated ESR	1.01 (1.00–1.01)	**0.002**		
BASDAI (baseline)	1.01 (1.01–1.01)	**< 0.001**		
BASFI (baseline) (median)	1.01 (1.01–1.01)	**< 0.001**	1.01 (1.00–1.01)	**0.001**
VAS global (baseline) (median)	1.01 (1.01–1.01)	**< 0.001**		
VAS pain (baseline)	1.01 (1.01–1.02)	**< 0.001**	1.01 (1.00–1.01)	**< 0.001**

Significant values are in [bold].

*CI* confidence interval, *ESR* erythrocyte sedimentation rate, *YoAS* young onset ankylosing spondylitis (symptom onset < 45 years), *LoAS* late onset ankylosing spondylitis (symptom onset ≥ 45 years), *BASDAI* bath ankylosing spondylitis disease activity index, *BASFI* bath ankylosing spondylitis functional index, *VAS* visual analog scale, *n* number, *%* percent.

**Table 5:** Factors associated with first TNFi discontinuation in YoAS and LoAS groups.

	YoAS				LoAS			
	HR (95% CI)	*p* value	HR (95% CI)	*p* value	HR (95% CI)	*p* value	HR (95% CI)	*p* value
Age, years (median)	1.00 (0.99–1.00)	0.852		–	0.98 (0.95–1.02)	0.499		–
Male (male vs. female)	0.63 (0.54–0.73)	**< 0.001**	0.63 (0.40–1.01)	0.052	0.69 (0.43–1.10)	0.122		–
Current smoker	0.87 (0.74–1.01)	0.071		–	1.24 (0.67–2.29)	0.486		–
HLA B27 (+)	0.75 (0.63–0.89)	**0.001**		–	0.43 (0.25–0.75)	**0.003**	0.56 (0.29–1.05)	0.072
Elevated ESR	1.01 (1.00–1.02)	**0.001**		–	1.00 (0.99–1.01)	0.870		–
BASDAI (baseline)	1.01 (1.00–1.02)	**< 0.001**			1.01 (1.00–1.02)	0.008		–
BASFI (baseline)	1.01 (1.00–1.02)	**< 0.001**	1.01 (1.01–1.019)	0.037	1.01 (1.00–1.02)	**0.003**		–
VAS global (baseline)	1.01 (1.00–1.02)	**< 0.001**		–	1.01 (1.00–1.02)	**0.004**	0.97 (0.95–1.01)	0.051
VAS pain (baseline)	1.01 (1.00–1.02)	**< 0.001**		–	1.02 (1.01–1.03)	**< 0.001**	1.04 (1.01–1.06)	**< 0.001**

Significant values are in [bold].

*HR* hazard ratio, *TNFi* tumor necrosis alpha inhibitor, *CI* confidence interval, *ESR* erythrocyte sedimentation rate, *YoAS* young onset ankylosing spondylitis (symptom onset < 45 years), *LoAS* late onset ankylosing spondylitis (symptom onset ≥ 45 years), *BASDAI* bath ankylosing spondylitis disease activity index, *BASFI* bath ankylosing spondylitis functional index, *VAS* visual analog scale, *n* number, *%* percent.

*Covariates with a *p* value < 0.1 in the univariable analysis included for multivariable analysis. Log-rank analysis, Cox regression model.

## Discussion

In this study, we demonstrated that real-life data from the TURKBIO Registry revealed comparable treatment responses to the first TNFi in 281 biologic naive patients with LoAS when compared to 2573 patients with YoAS. Moreover, we observed similar overall retention rates between the LoAS and YoAS groups, irrespective of the type of TNFi used. Notably, our comprehensive literature review highlighted the scarcity of studies investigating LoAS patients, with only one study, conducted by Kim et al.^19^, evaluating treatment response and retention rates for biologics in this patient subgroup. Their study, based on data from the Korean Biological Database (KOBIO), assessed 236 LoAS patients with disease onset at the age of 50 years or older among a total of 1708 AS patients. Kim et al. reported that LoAS patients exhibited lower treatment response and retention rates in comparison to YoAS individuals. LoAS was inversely associated with achieving desired outcomes such as ASDAS-CII, ASAS 20, and ASAS 40 responses. Additionally, the change in BASDAI at the 1-year mark was less pronounced in LoAS patients. However, it's worth noting that ESR and CRP demonstrated a more substantial decline in LoAS, while there were no significant differences in the changes related to patient global assessment, ASDAS-CRP, ASDAS-ESR, and BASFI between the two groups during the 1-year follow-up. Notably, no association was found between LoAS and discontinuation of bDMARDs^19^.

It is widely known that methodological disparities can contribute to variations in study outcomes. In our investigation, we applied a cut-off age criterion of ≥ 45 years at disease onset for the classification of LoAS, aligning with the ASAS AxSpA classification criteria^20^. In contrast, Kim et al.^19^ opted for a cut-off age of ≥ 50 years at onset in their research. Importantly, another study has indicated that individuals with an age of onset falling within the range of 45–50 years comprised 25% of the overall LoAS group, introducing potential diversity in the study findings^20^. In addition, factors such as gender, age, duration of disease before starting treatment, as well as geographic and racial characteristics, can introduce disparities in treatment response and retention rates across different studies.

Using predefined sets of diagnostic criteria and standardized clinical and radiographic indicators of disease activity, we obtained data from a national registry for this study. Consequently, our cohort of 281 patients with LoAS and 2573 patients with YoAS constituted a substantial real-world dataset, thereby serving the primary objectives of our study. An additional strength of our research lies in our focus on the response to the first TNFi treatment in biologic-naïve patients with both LoAS and YoAS, in contrast to Kim et al.’s study, which evaluated the entire spectrum of biologic treatment series^19^. By doing so, we mitigated potential confounding factors, such as the changing treatment responses associated with multiple treatment series.

As far as we are aware, this research is the first to provide a thorough analysis of the safety and effectiveness of TNFi medication in patients with LoAS as opposed to YoAS patients. Leveraging a substantial real-world dataset with extended follow-up durations allowed us to ascertain AEs during TNFi treatment. Notably, we observed no significant disparities in the frequency of AEs, and we did not identify any new safety signals in LoAS patients as compared to YoAS. This finding has important implications as it may alleviate the concerns of rheumatologists regarding potential increases in AEs when introducing TNFi treatment to patients with LoAS.

The classification of patients with AS who develop symptoms at an advanced age remains a subject without a universally accepted consensus. The criterion of onset before 45 years of age for chronic back pain in the ASAS AxSpA classification criteria^21^ may limit their applicability in studies involving LoAS patients. Consequently, it is currently suggested that the mNY criteria, which do not impose an age limit, be employed to standardize studies on LoAS. Bendahan et al.^8^ recently demonstrated that the performance of the ASAS AxSpA classification criteria improved after accommodating for age, surpassing the Amor and ESSG criteria for axSpA. All of the LoAS patients in this research satisfied the mNY requirements for AS, and when the ASAS AxSpA criteria were adjusted to incorporate an age of onset for back pain of ≥ 45 years, they also satisfied the radiographic AxSpA criteria.

The reported prevalence of LoAS within the broader AS patient population has exhibited variability, ranging from 3.5 to 9.7% across studies that employed different cut-off values such as 40, 45, or 50 years at onset^3,7,22^. In our investigation, we observed that LoAS patients accounted for 9.9% of the 2854 AS patients who received their initial TNFi treatment as part of the TURKBIO Registry. Notably, the frequency of LoAS was higher (13.8%) in the KOBIO Registry, compared to our findings. However, it’s essential to highlight that the KOBIO Registry included patients with disease onset at ≥ 50 years^19^.

In patients with AS, male predominance and HLA-B27 positivity tend to decrease in late onset groups^5,7^. Consistent with findings from the KOBIO Registry^19^, this study also identified a lower frequency of male patients and HLA-B27 positivity in LoAS patients who initiated their first TNFi treatment compared to YoAS patients. At baseline, each measure of disease activity was comparable between the groups, except for ESR, which was found to be higher in LoAS patients, potentially influenced by their older age. Despite the shorter disease duration before TNFi treatment, LoAS patients exhibited higher baseline BASFI and BASMI scores. Since the study included only patients receiving their initial TNFi, these results cannot be generalized to all LoAS patients. However, the similarity in baseline values suggests that physicians did not require higher disease activity levels in LoAS patients compared to YoAS patients to initiate the first biologic treatment. This similarity in baseline disease activity levels eliminated bias when comparing treatment responses based on binomial measures, including BASDAI50, ASDAS-ID, ASDAS-MI, and ASDAS CII.

In this study, there was no significant difference in the occurrence of axial and peripheral joint involvement between the LoAS and YoAS groups, which aligns with the findings of a previous study conducted in Turkey^23^. However, it's worth noting that other cohorts, such as those in Spain^4^ and South Korea^19^, reported a heightened prevalence of peripheral arthritis in LoAS patients. Interestingly, Japanese patients with late-onset SpA exhibited an increased frequency of tenosynovitis, peripheral enthesitis, and synovitis compared to their early-onset counterparts, particularly in cases where the prevalence of SpA and HLA-B27 was notably low^24^.

The groups in this research had comparable baseline NSAID usage frequencies. However, more LoAS patients were using corticosteroids and csDMARD compared to YoAS at baseline, even though there was no increased frequency of peripheral involvement. This might suggest that physicians prefer to initiate a trial of corticosteroids and csDMARD before deciding on TNFi treatment, possibly due to concerns about the adverse effects of NSAIDs in older patients. Similarly, data from Brazil^8^ showed increased use of corticosteroids and csDMARDs in LoAS patients compared to YoAS, even though there were no differences in the frequencies of peripheral involvement, NSAID use, and biologic treatment. The use of NSAIDs in the treatment of AS is regulated in accordance with guidelines, regardless of age groups. Therefore, it remains the primary pharmacological treatment option in young or elderly AS patients in cases where there are no clinical contraindications. Since we could not evaluate the side effects associated with NSAID use in AS patients in our cohort results, an intergroup evaluation could not be made.

This study is not without its limitations. Firstly, the data are drawn from a registry, which may introduce the potential for selection bias and incomplete information. Additionally, the study primarily focuses on the Turkish population, and the results may not be entirely generalizable to other populations due to potential geographic and racial differences. Secondly, the follow-up duration of over 24 months may not capture very long-term effects or rare AEs associated with TNFi therapy. Finally, the study does not delve into specific TNFi agents, which may have differing effects on YoAS and LoAS patients.

It's crucial to recognize this study's advantages, too, though. The use of a nationwide registry allowed for the collection of real-life data from a large and diverse patient population. This contributed to robust statistical analyses and increased the generalizability of the findings to real-world clinical settings. Furthermore, the study uniquely focused on TNFi treatment in biologic naïve LoAS and YoAS patients, eliminating potential confounding factors related to prior biologic treatment series. The extensive follow-up duration provides valuable insights into the long-term outcomes and safety of TNFi therapy in these patients. Finally, it was observed that LoAS patients did not develop a remarkable side effect profile with TNFi treatment in our cohort's long-term follow-up data.

In conclusion, this real-life study demonstrates that initial TNFi treatment in patients with YoAS and LoAS results in similar response and retention rates, despite differences in gender distribution and HLA-B27 positivity between the groups. Furthermore, it reveals no additional risk of AEs with TNFi treatment in LoAS patients, even after a follow-up period of more than 24 months, when compared to YoAS. These findings suggest that the management of LoAS patients with TNFi therapy can be similar to that of YoAS patients. Considering the potentially higher risk of AEs related to NSAID use in LoAS patients, it is advisable to proceed with TNFi therapy when it is warranted, provided that it is accompanied by proper indications and diligent follow-up. To validate these results, further engagement of LoAS patients in clinical and epidemiological studies is essential.

## Data Availability

The datasets used and/or analysed during the current study available from the corresponding author on reasonable request.
